# Self-medication with antibiotics for the treatment of menstrual symptoms in southwest Nigeria: a cross-sectional study

**DOI:** 10.1186/1471-2458-10-610

**Published:** 2010-10-15

**Authors:** Amy R Sapkota, Morenike E Coker, Rachel E Rosenberg Goldstein, Nancy L Atkinson, Shauna J Sweet, Priscilla O Sopeju, Modupe T Ojo, Elizabeth Otivhia, Olayemi O Ayepola, Olufunmiso O Olajuyigbe, Laura Shireman, Paul S Pottinger, Kayode K Ojo

**Affiliations:** 1 Maryland Institute for Applied Environmental Health, University of Maryland College Park, School of Public Health, College Park, MD, USA; 2 University of Ibadan, Ibadan, Oyo State, Nigeria; 3 Department of Public and Community Health, University of Maryland College Park, School of Public Health, College Park, MD, USA; 4 Olabisi Onabanjo University, Ago-Iwoye, Ogun State, Nigeria; 5 Covenant University, Ota, Ogun State, Nigeria; 6 Babcock University, Ikeja, Ogun State, Nigeria; 7 University of Washington, Seattle, WA, USA

## Abstract

**Background:**

Self-medication with antibiotics is an important factor contributing to the development of bacterial antibiotic resistance. The purpose of this study was to evaluate the prevalence of self-medication with antibiotics for the treatment of menstrual symptoms among university women in Southwest Nigeria.

**Methods:**

A cross-sectional survey was administered to female undergraduate and graduate students (n = 706) at four universities in Southwest Nigeria in 2008. The universities were selected by convenience and the study samples within each university were randomly selected cluster samples. The survey was self-administered and included questions pertaining to menstrual symptoms, analgesic and antibiotic use patterns, and demographics. Data were analyzed using descriptive statistics and logistic regression.

**Results:**

The response rate was 95.4%. Eighty-six percent (95% CI: 83-88%) of participants experienced menstrual symptoms, and 39% (95% CI: 36-43%) reported using analgesics to treat them. Overall, 24% (95% CI: 21-27%) of participants reported self-medicated use of antibiotics to treat the following menstrual symptoms: cramps, bloating, heavy bleeding, headaches, pimples/acne, moodiness, tender breasts, backache, joint and muscle pain. Factors associated with this usage were: lower levels of education (Odds Ratio (OR): 2.8, 95% CI: 1.1-7.1, *p-*value: 0.03); non-science major (OR: 1.58, 95% CI: 1.03-2.50, *p*-value: 0.04); usage of analgesics (OR: 3.17, 95% CI: 2.07-4.86, *p*-value: <0.001); and mild to extreme heavy bleeding (OR: 1.64, 95% CI: 1.01-2.67, *p*-value: 0.05) and pimples/acne (OR: 1.57, 95% CI: 0.98-2.54, *p*-value: 0.06). Ampicillin, tetracycline, ciprofloxacin and metronidazole were used to treat the most symptoms. Doctors or nurses (6%, 95% CI: 4-7%), friends (6%, 95% CI: 4-7%) and family members (7%, 95% CI: 5-8%) were most likely to recommend the use of antibiotics for menstrual symptoms, while these drugs were most often obtained from local chemists or pharmacists (10.2%, 95% CI: 8-12%).

**Conclusions:**

This is the first formal study to report that approximately 1 out of 4 university women surveyed in Southwest Nigeria self-medicate with antibiotics to treat menstrual symptoms. This practice could provide monthly, low-dose exposures to antibiotics among users. Further studies are necessary to evaluate the impacts of self-medication on student health.

## Background

Increasing rates of antimicrobial resistance have left clinicians with limited drug options for the treatment of bacterial infectious diseases. This is a major public health concern worldwide, especially in developing countries where higher rates of resistant bacterial infections persist [1,2]. For example, rates of multiple antibiotic resistance among urinary tract infection (UTI) bacterial isolates in Southwest Nigeria are significantly higher than that of any other country [3]. In addition, rates of other life-threatening bacterial infections, such as community-acquired methicillin-resistant *Staphylococcus aureus *(MRSA), continue to rise in African countries including Nigeria [4] and Botswana [5]. Because the misuse and abuse of antibiotics is a major cause of antimicrobial resistance, research is needed to evaluate the specific antibiotic usage patterns that are prevalent in developing countries so that interventions can be developed and implemented. For such interventions to be effective, we must also understand the underlying socio-cultural factors that contribute to antimicrobial misuse and the subsequent amplification of resistance in human populations [1,2].

In Nigeria, there are limited controls on the sale or advertisement of antimicrobials, creating opportunities for misinformation and misperceptions that can exacerbate improper antibiotic use [1,6]. In addition, counterfeit drugs and poor pharmaceutical qualities of available antimicrobials (containing no or substandard active ingredients) have been widely reported [7-9]. These factors often lead to higher rates of resistance to less-expensive first-line regimens compelling subsequent changes in treatment protocols to include more expensive and sometimes more toxic drugs [10]. In addition, access to good and effective medical interventions is often limited due to poor hospital facilities; service fees; poverty and hunger; and illiteracy [1,2,6,11]. Patronage of "quacks," untrained individuals providing unconventional and unhygienic medical care, is therefore widespread and frequently becomes institutionalized as normal.

Within this paradigm, self-medication--previously defined by The World Health Organization [12]--is widely encouraged, even among educated elites, as a justification for preserving the scarce resources of trained physicians and other medical personnel [13]. Recent studies have sought to understand patterns of self-medication with antibiotics in developing and other countries [13-17]. These studies have identified several indications for self-medication with antibiotics including the common cold, [13,14,16] diarrhea or constipation,[15] and sore throat [16]. In a pilot survey that we conducted in July 2007 in Ago-Iwoye, Lagos, and Ibadan, Nigeria, we observed a disturbing new trend among university women of self-medication with antibiotics to treat menstrual symptoms [6]. In particular, participating women reported using antibiotics to reduce cramps, regulate heavy flow and prevent "infections" from feminine sanitary products [6]. However, comprehensive data regarding the magnitude of antibiotic self-medication for menstrual symptoms, the specific "other" menstrual symptoms that were being "treated," and the characteristics of the women who were more likely to self-medicate were not collected.

Therefore, the purpose of the present study was to determine the prevalence of self-medication with antibiotics for the treatment of menstrual symptoms among university women in Southwest Nigeria and to evaluate factors associated with this practice. Our pilot study [6] and a recent study conducted by Afolabi in Lagos State, Nigeria [18], informed the selection of factors evaluated in the present study.

## Methods

### Survey Setting

This study was carried out in February 2008 at four universities (two public and two private universities) located in Southwest Nigeria. The public universities were the University of Ibadan (>12,000 total students) located in Oyo State and Obafemi Awolowo University (25,000 total students) located in Osun State. The private universities were Covenant University (6,000 total students) and Babcock University (6,000 total students), which are both located in Ogun State. These universities were selected via convenience sampling and were included in the study because students attending public versus private universities often represent different socioeconomic groups and we sought to evaluate whether socioeconomic status is one potential factor that influences the use of antibiotics for menstrual symptoms among university women.

The four universities included in the study account for 12% of the 34 operational universities in Southwest Nigeria which are comprised of 6 federally-funded universities, 10 state-funded universities, and 18 privately-owned universities [19]. These 34 universities represent 33% of the total 104 Nigerian universities which are comprised of 27 federally-funded universities, 36 state-funded universities and 41 privately-owned universities throughout the country [20].

Approximately 69% of Nigerians complete primary school [21], and a World Bank report published in 2000 estimated that higher education in Nigeria only enrolls about 4% of the eligible age cohort [22]. A recent report described that the proportion of female students attending Nigerian higher institutions is rising to about 47% of overall students [19]. The proportion of female students at the universities included in this study was approximately 50%.

### Sample size calculation

A sample size calculation was performed using the following equation: n = (Z^2 ^*P*(1-*P*))/(*d*^2^), where n = sample size, Z = Z statistic corresponding to a chosen level of confidence, *P *= expected prevalence, and *d *= precision [23]. In our calculation, we used Z = 1.96, *P *= 0.3 and *d = *0.05. This calculation resulted in a sample size of 323. This sample size was doubled to account for the clustered nature of the study design [24,25], resulting in a sample size of 646. To account for non-responses, researchers typically increase the calculated sample size by anywhere from 5 to 20% [24,25]. We increased the sample size by 5% to account for non-responses, resulting in a sample size of 679. However, to be more conservative, we cushioned our sample size by an additional (arbitrary) ~10%, arriving at a total sample size, n = 740. This total sample size was divided by the number of clusters (4 universities) included in the study to determine how many surveys should be administered at each university [25,26]. This method of dividing the sample equally among clusters was in accordance with "generic cluster sample" design methods previously described by the WHO Department of Vaccines and Biologicals [26].

### Sampling strategy

At the University of Ibadan, Covenant University and Obafemi Awolowo University, study participants were recruited from residence halls. The sampling strategy at these universities was a three-stage cluster sampling plan. Female residence halls that housed both undergraduate and graduate students were randomly selected for inclusion in the study. Within the residence halls, blocks of rooms were then randomly selected for inclusion in the study and every resident of those rooms was invited to participate in the survey.

At Babcock University, study participants were recruited from lecture halls because we were ultimately not granted access to the residence halls at this university. Thus, at Babcock University, a one-stage cluster sampling strategy was employed. Lecture halls that housed both undergraduate and graduate classes were randomly selected for participation in the study and every female student within the randomly selected lecture halls was invited to participate in the study.

Institutional review board (IRB) approval was granted by the University of Maryland College Park IRB. Written informed consent was obtained from all study participants. No incentive was offered for completion of the survey.

### Survey Instrument

Qualitative interviews conducted among twenty-seven young Nigerian women who anecdotally reported using antibiotics to normalize menstrual flow and treat discomfort associated with menstruation [6] informed the initial content and focus of the survey. A preliminary version of the survey was then piloted among a focus group of nine female instructors and students at the University of Ibadan to evaluate language, content, and sensitivity of the instrument [27]. Overall, focus group participants indicated that the survey was "good," "straight forward," and "important." However, focus group participants did suggest that a few minor language edits should be incorporated into the final survey. These edits were incorporated and the revised survey instrument was also visually reformatted to improve flow and conceptual clarity [28].

The final survey instrument (Additional file 1) consisted of four sections containing both closed and open-ended questions. The first section included questions concerning menstrual periods, in particular pain and other symptoms experienced during menstruation. The second section consisted of questions relating to analgesics and antibiotics that women may have taken before, during, or after menstruation. The third section contained general demographic questions, and the fourth section consisted of detailed demographic questions relating to family status (e.g. single, married, etc.) and sexual activity. Responses to three questions in the second section (Questions 11, 12 and 13 shown in Additional file 1) were used to calculate the prevalence of recent antibiotic usage for menstrual symptoms. If a participant answered "Yes" to question 11 or checked off any box in questions 12 or 13, they were considered a "user" of antibiotics for the treatment of menstrual symptoms. "Self-medication" with antibiotics was verified using the participants qualitative answers to the open-ended question at the end of the questionnaire (Question 22 shown in Additional file 1).

### Administration of Survey Instrument

At each university, the surveys were administered by our Nigerian collaborators, who are instructors, lecturers or affiliates at the participating universities. All survey administrators used the same script and study protocol to invite participants into the study, obtain informed consent, and administer the surveys.

### Statistical Analyses

The survey data were checked, coded, and entered into a Microsoft Access database. The data were then cleaned and analyzed using descriptive and inferential statistics. Simple and multiple logistic regression models were used to evaluate associations between participant characteristics and reported usage of antibiotics to treat menstrual symptoms. All statistical analyses were carried out using Stata/IC 10 (StataCorp, College Station, Texas). Continuous data are presented as means, along with their 95% confidence intervals (CIs).

## Results

### Study population characteristics

A total of 706 out of 740 administered surveys (95.4% response rate) were completed and returned by female students attending Babcock University, Covenant University, University of Ibadan, and Obafemi Awolowo University. The characteristics of the study population are summarized in Table 1.

**Table 1:** Study population (n = 706) characteristics

Demographic Characteristics	n	%
University		
Babcock University	237	33.6
Covenant University	167	23.7
University of Ibadan	149	21.1
Obafemi Awolowo University	153	21.7

Education Level		
Preliminary level (Pre-college)	3	0.42
Freshman (100 level)	166	23.5
Sophomore (200 level)	194	27.5
Junior (300 level)	171	24.2
Senior (400 level)	73	10.3
Final year of Pharmacy, Law, or Engineering	45	6.4
Final year of Medical School	2	0.3
Masters or Doctorate	26	3.7
Missing data	26	3.7

Area of Concentration		
Lab Science, Medicine	285	40.4
Social Sciences	135	19.1
Humanities (Arts, Languages)	97	13.7
Business and Finance	102	14.5
Technology	53	7.5
Missing data	35	5.0

Age		
< 17	16	2.3
17-20	330	46.7
21-24	250	35.4
25-29	66	9.4
30-34	16	2.3
35-39	6	0.9
≥ 40	1	0.1
Missing data	21	3.0

Marital Status		
Single	609	86.3
Engaged, Married, Separated,		
Divorced or Widowed	74	10.5
Missing data	23	3.3

### Menstruation

The majority of study participants (88%: 95% CI, 86% to 91%) reported experiencing three or four menstrual periods during the three-month time period that served as the focus for this study. Eighty-six percent (95% CI: 83% to 88%) of participants reported experiencing pain or discomfort associated with their menstrual periods during this time frame. Yet, only 21% (95% CI: 18% to 24%) of study participants reported ever having seen a doctor or nurse for pain or discomfort associated with their menstrual periods. The specific types of pain or discomfort that were experienced included cramps; weight gain and water retention; heavy flow; headaches; pimples or acne; moodiness; tender or painful breasts; backache, joint or muscle pain; and other symptoms.

### Prevalence of self-medication

When asked about the self-medicated use of analgesics and other pain-relieving medications, such as aspirin and ibuprofen, 39% (95% CI: 36% to 43%) of respondents reported that they had used these types of medications to treat menstrual symptoms in the past three months. The usage prevalence for these drugs was as follows: aspirin, 2% (95% CI: 1% to 3%); Panadol (paracetamol), 29% (95% CI: 26% to 33%); Panadol Extra (paracetamol and caffeine), 7% (95% CI: 5% to 9%); ibuprofen, 9% (95% CI: 7% to 12%); buscopan (butylscopolamine), 10% (95% CI: 8% to 12%); Feldene (piroxicam), 11% (95% CI: 8% to 13%); and other drugs, 11% (95% CI: 8% to 13%). These other drugs included, but were not limited to, codeine, Midol, Tylenol and Advil.

**Figure 1:**
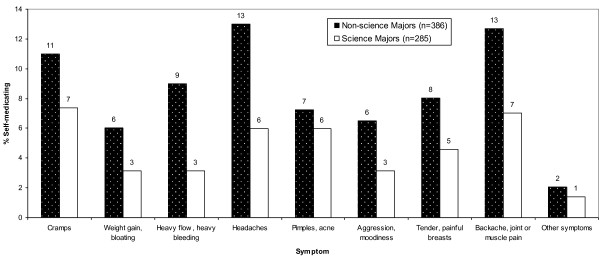
**Prevalence of self-medication with antibiotics for the treatment of menstrual symptoms among University women in Southwest Nigeria by major area of study and symptoms**.

Overall, 24% (95% CI: 21% to 27%) of the study population reported self-medicating with antibiotics to treat menstrual symptoms in the past three months. The mean age when study participants first started taking antibiotics to treat menstrual symptoms was 15.7 ± 2.96 years. Interestingly, the prevalence of self-medicated antibiotic use for menstrual symptoms varied depending on education level (Table 2). In addition, individuals who were non-science majors were more likely to use antibiotics for menstrual symptoms compared with lab science, public health or medicine majors (Table 2, Figure 1). Study participants who used any pain-relieving medications (e.g. aspirin, ibuprofen) to treat menstrual symptoms were more likely to use antibiotics to treat menstrual symptoms than those who did not use any pain relievers (Table 2).

**Table 2:** Results of multivariate analysis of factors that may influence self-medication with antibiotics for the treatment of menstrual symptoms.

Independent Variable	Odds Ratio	95% Confidence Interval	*p- *value
Cramps			
No (n = 187)	1.00	-	-
Yes (Mild to severe) (n = 435)	0.57	(0.36 - 0.91)	0.02
Heavy flow/heavy bleeding			
No (n = 186)	1.00	-	-
Yes (Mild to severe) (n = 436)	1.64	(1.01-2.67)	0.05

Pimples/acne			
No (n = 182)	1.00	-	-
Yes (Mild to severe) (n = 456)	1.57	(0.98-2.54)	0.06
Education level			
Preliminary and undergraduate levels (n = 607)	1.00	-	-
Graduate level (n = 73)	0.36	(0.14 - 0.91)	0.03

Major			
Lab science, public health or medicine (n = 285)	1.00	-	-
Non-science (n = 386)	1.58	(1.03 - 2.50)	0.04

Use of pain relievers to relieve menses symptoms			
No (n = 411)	1.00	-	-
Yes (n = 276)	3.17	(2.07 - 4.86)	<0.001

Age			
≤ 20 (n = 346)	1.00	-	-
21-29 (n = 316)	1.09	(0.68-1.74)	0.72
≥ 30 (n = 23)	1.78	(0.31-10.1)	0.52

University			
Babcock University (n = 237)	1.00	-	-
Covenant University (n = 167)	1.13	(0.62-2.07)	0.68
University of Ibadan (n = 149)	1.61	(0.90-2.88)	0.10
Obafemi Awolowo University (n = 153)	1.26	(0.69-2.28)	0.45

In terms of specific symptoms, those study participants who reported mild to severe heavy flow/heavy bleeding and mild to severe pimples/acne associated with menstruation were more likely to use antibiotics to treat these symptoms compared to individuals who reported experiencing no heavy flow/heavy bleeding or pimples/acne (Table 2). Surprisingly, women who reported mild to severe cramps were less likely to use antibiotics compared with those who reported experiencing no cramps (Table 2). Factors including age, specific university attended (hence, socio economic status), marital status, and sexual activity were not significantly associated with self-medicated use of antibiotics to treat menstrual symptoms.

### Types of antibiotics used

Study participants reported using 12 antibiotics to treat a variety of symptoms before, during, and after menses. However, nearly all antibiotic users reported using only one antibiotic for the treatment of either one or more specific symptoms during the 3-month period that served as the focus for the survey. Table 3 summarizes the types of antibiotics that were used to treat specific menstrual symptoms and provides estimates of the prevalence of use for each antibiotic. Ampicillin, tetracycline, ciprofloxacin and metronidazole were used to treat the most menstrual symptoms (≥ 7 symptoms). If a preferred antibiotic was not available, 8% (95% CI: 6% to 10%) of study participants reported that they would use another type of antibiotic to treat the specific menstrual symptom. When asked whether these antibiotics were effective in relieving symptoms, a number of participants reported that the drugs relieved each of the symptoms, of which the largest proportions indicated that antibiotics relieved backache, joint or muscle pain (10%, 95% CI: 8% to 12%), headaches (10%, 95% CI: 8% to 12%), and cramps (9%, 95% CI: 6% to 11%).

**Table 3:** Number of study participants using specific antibiotics to treat specific menstrual symptoms (n, (%, 95% confidence interval)), and estimates of the overall prevalence of use for each antibiotic for one or more menstrual symptoms (%, 95% confidence interval)

					Symptoms					
Antibiotic	Cramps	Weight gain, bloating	Heavy flow, heavy bleeding	Headaches	Pimples, acne	Aggression, moodiness	Tender, painful breasts	Backache, joint or muscle pain	Other	Prevalence of use for ≥ 1 menstrual symptom
Ampicillin	4 (0.6, 0.01-1.1)	5 (0.7, 0.08-1.3)	6 (0.9, 0.1-1.5)	2 (0.3, 0.1-0.6)	2 (0.3, 0.1-0.6)	0	1 (0.14, -0.01-0.4)	2 (0.3, 0.1-0.6)	3 (0.4, 0.05-0.9)	2.6 (1.4-3.7)
Ampiclox (Ampicillin + Cloxacillin)	2 (0.3, 0.1-0.6)	0	6 (0.9, 0.1-1.5)	1 (0.14, -0.01-0.4)	2 (0.3, 0.1-0.6)	0	0	0	1 (0.14, -0.01-0.4)	2.0 (0.95-3.0)

Pefloxacin	1 (0.14, -0.01-0.4)	3 (0.4, 0.05-0.9)	1 (0.14, -0.01-0.4)	0	0	0	0	1 (0.14, -0.01-0.4)	0	0.9 (0.2-1.5)

Amoxicillin	2 (0.3, 0.1-0.6)	1 (0.14, -0.01-0.4)	3 (0.4, 0.05-0.9)	3 (0.4, 0.05-0.9)	0	0	0	0	0	1.3 (0.4-2.1)

Augmentin	1 (0.14, -0.01-0.4)	1 (0.14, -0.01-0.4)	2 (0.3, 0.1-0.6)	0	0	0	0	0	1 (0.14, -0.01-0.4)	0.7 (0.1-1.3)

Ofloxacin	2 (0.3, 0.1-0.6)	1 (0.14, -0.01-0.4)	2 (0.3, 0.1-0.6)	2 (0.3, 0.1-0.6)	0	0	0	0	0	1.1 (0.4-1.9)

Tetracycline	6 (0.9, 0.1-1.5)	1 (0.14, -0.01-0.4)	4 (0.6, 0.01-1.1)	1 (0.14, -0.01-0.4)	0	0	1 (0.14, -0.01-0.4)	2 (0.3, 0.1-0.6)	7 (1.0, 0.2-1.7)	2.7 (1.5-3.9)

Ciprofloxacin	2 (0.3, 0.1-0.6)	1 (0.14, -0.01-0.4)	1 (0.14, -0.01-0.4)	2 (0.3, 0.1-0.6)	1 (0.14, -0.01-0.4)	0	0	1 (0.14, -0.01-0.4)	4 (0.6, 0.01-1.1)	1.4 (0.5-2.3)

Metronidazole	6 (0.9, 0.1-1.5)	1 (0.14, -0.01-0.4)	2 (0.3, 0.1-0.6)	1 (0.14, -0.01-0.4)	0	1 (0.14, -0.01-0.4)	1 (0.14, -0.01-0.4)	1 (0.14, -0.01-0.4)	1 (0.14, -0.01-0.4)	3.0 (1.7-4.2)

Cotrimoxazole	3 (0.4, 0.05-0.9)	0	0	3 (0.4, 0.05-0.9)	1 (0.14, -0.01-0.4)	0	1 (0.14, -0.01-0.4)	1 (0.14, -0.01-0.4)	2 (0.3, 0.1-0.6)	1.6 (0.6-2.5)

Streptomycin	1 (0.14, -0.01-0.4)	0	2 (0.3, 0.1-0.6)	1 (0.14, -0.01-0.4)	0	0	0	0	0	0.6 (0.01-1.1)

Gentamicin	3 (0.4, 0.05-0.9)	1 (0.14, -0.01-0.4)	0	2 (0.3, 0.1-0.6)	1 (0.14, -0.01-0.4)	0	0	0	0	1.0 (0.3-1.7)

### Sources of information and antibiotics

**Figure 2:**
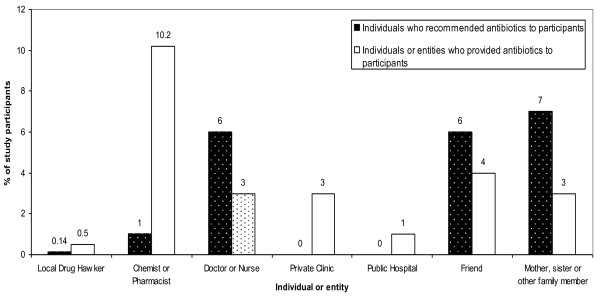
**Individuals who recommended the use of antibiotics to treat menstrual symptoms and individuals or entities who provided antibiotics to study participants**.

Study participants also reported that a variety of individuals first recommended that they take antibiotics to treat menstrual symptoms. Doctors or nurses (6%, 95% CI: 4% to 7%), friends (6%, 95% CI: 4% to 7%) and family members (7%, 95% CI: 5% to 8%) were the individuals who were most often cited as recommending antibiotics for these symptoms (Figure 2). However, the antibiotics used to treat menstrual symptoms were most often obtained from local chemists or pharmacists (10.2%, 95% CI: 8% to 12%) (Figure 2).

## Discussion

In this study, we identified that approximately 1 out of 4 surveyed female university students in Southwest Nigeria practiced self-medication with antibiotics to treat a variety of menstrual symptoms. Ampicillin, tetracycline, ciprofloxacin and metronidazole were used to treat the most menstrual symptoms, and the drugs were most often obtained from local chemists or pharmacists. To our knowledge, this is the first formal study to report such a high rate of antibiotic self-medication among women of child-bearing age in Southwest Nigeria.

These results are potentially more alarming compared with other unorthodox, self-medicated uses of antibiotics because the menstrual cycle occurs monthly. While we did not collect data concerning the frequency of self-medication with antibiotics for the treatment of menstrual symptoms, we speculate that the use of antibiotics for menstrual symptoms that may last only a few days every month could possibly provide frequent, low-dose exposures among users. Low doses of antibiotics on a regular basis may expose the normal gut bacterial flora--which can cause UTIs--to sub-inhibitory doses that favor the selection of resistant bacteria. A previous study in Southwest Nigeria identified a high prevalence of UTIs among women of child-bearing age [29], and other studies have shown that the prevalence of multiple resistance to commonly-used antibiotics among UTI bacterial isolates from Southwest Nigeria is considerably higher than that of any other part of the world [3,30-32]. Thus, it is possible that the self-medication behaviors that we have identified among young women in this study may be contributing to the elevated rates of antibiotic-resistant UTIs that have been documented in this region.

However, additional studies are necessary to identify whether the antibiotic self-medication practices described here are also prevalent among Nigerian women who do not attend college. The young women surveyed in this study represent a small, elite proportion of the female, Nigerian population [21]. Thus, it is unclear whether the responses of the surveyed women are representative of other women in Southwest Nigeria (and beyond) who do not attend college. Similarly, at this time, it is difficult to estimate 1) the overall magnitude of the antibiotic selective pressures; and 2) the specific subsequent effects on bacterial antibiotic resistance that are associated with self-medicated antibiotic usage for the treatment of menstrual symptoms in Nigeria.

While the present study was the first formal study to assess self-medication with antibiotics for menstrual symptoms among university women in Southwest Nigeria, another recent study identified antibiotic use for menstrual symptoms among secondary school girls in Osun State (the location of one of our participating universities) [33]. In this study, Ogunfowokan and Babatunde (2010) described that 1% of secondary school girls reported using tetracycline for the management of menstrual pain [33]. This is similar to our finding that 2.7% of our study participants specifically used tetracycline to treat several menstrual symptoms including cramps, and heavy flow (Table 3). However, since Ogunfowokan and Babatunde (2010) used a survey with open-ended questions on the self-management of menstrual symptoms and tetracycline was noted as the only cited antibiotic, the overall prevalence of antibiotic use for the treatment of menstrual symptoms is likely lower in the Ogunfowokan and Babatunde (2010) study compared to the present study.

Beyond self-medicated use of antibiotics for menstrual symptoms, other studies have evaluated antibiotic self-medication patterns, in general, among other populations in Nigeria indicating that this overall practice is common. For example, Afolabi et al. (2010) recently identified that 30.4% of surveyed dental patients in Ondo State-- which is also located in Southwest Nigeria--practiced self-medication with antibiotics [34]. In another recent study, Afolabi (2008) assessed factors that are associated with self-medication, in general, among market women (mostly of the Yoruba ethnic group) in Lagos State, located in Southwest Nigeria [18]. In this study, level of education was identified as a major factor that influenced self-medication patterns, while age was not significantly associated with self-medication [18]. These data are similar to our findings that show that higher levels of education are inversely associated with self-medicated antibiotic use for menstrual symptoms (Table 2), while age and socio-economic status are not significantly associated with this type of antibiotic usage. In contrast, Afolabi (2008) identified that market women most often obtained their information about medications, as well as the medications themselves, from patent medicine stores, while the present study found that information about antibiotic usage and the antibiotics themselves were most often obtained from family members and local chemists or pharmacists, respectively. Previous studies conducted in Africa have also identified pharmacies as important sources of self-administered drugs [17,35].

Understanding the sources of information and sources of drugs for antibiotic self-medication can help in the formulation of community-based interventions that can help to reduce self-medication practices. Our data suggest that interventions are indicated at several levels: public health education directed at populations, and medical education directed at health professionals. Since girls are more likely to receive information about menstruation and other health issues from their mothers, other female relatives and friends rather than through formal education sources [36,37], providing health education on the appropriate use of antibiotics to female family members in the general population may be more productive initially than educating university or school girls directly.

Medical education efforts also are indicated since our findings show that clinicians are likely to recommend and pharmacists are likely to provide antimicrobials for menstrual problems. These efforts could be directed at medical and other health profession students who are at the initial stages of their medical education, as well as practicing physicians, and other healthcare professionals. Encouragingly, we found that students who studied medicine and public health were less likely to misuse antibiotics for menstrual complaints compared to non-science majors. This suggests that those women who are currently studying disciplines related to healthcare fields could positively impact the individuals whom they treat in the future with regard to potential reductions in self-medication practices [15].

This is a cross-sectional study that utilized a self-administered survey to estimate the prevalence of self-medicated antibiotic use in the past. Therefore, by design, recall bias cannot be ruled out. In addition, since the survey was self-administered, respondents may have skipped questions that they did not understand. Moreover, respondents may have underreported antibiotic usage because issues related with menses are culturally sensitive and often viewed as a taboo subject. Respondents also might not have known what an antibiotic is; although this may be less of an issue particularly among our survey respondents since they were all university students. The fact that surveys were administered in residence halls or lecture halls depending on the university is also a limitation. However, the data show that survey administration in either residence halls or lecture halls was not a factor that influenced the findings.

Another limitation of the study is that, while random sampling was completed at each university, the universities themselves were selected by convenience. In a study of this type, we did not have the ability to randomly select a sample of all Nigerian universities for inclusion in the study because we did not have the luxury of having collaborators present at every Nigerian university who could complete the necessary field work. As a result, it is unclear whether our findings are generalizable to other Nigerian universities.

## Conclusions

In summation, our findings provide the first prevalence data on self-medication with antibiotics for the treatment of menstrual symptoms among young Nigerian university women. We speculate that this type of practice could provide monthly, low-dose exposures to antibiotics among users, and could partially explain the high rates of antibiotic-resistant UTIs previously described in Nigeria.

By targeting educated women of child-bearing age, this study addresses a population with more resources than the general population. Future research should be expanded to include other populations of Nigerian women to determine the overall prevalence of self-medicated antibiotic use for menstrual symptoms in the country, as well as any additional knowledge deficits and attitudinal barriers to eliminating antimicrobial misuse. Such information could then be used to develop education initiatives and theory-based behavior modification programs directed at reducing the misuse of antibiotics among women and their healthcare providers in Nigeria.

## Competing interests

The authors declare that they have no competing interests.

## Authors' contributions

ARS conceived, designed and supervised the study; performed the data analysis; and led the writing. MOC helped in the design of the study and study instrument; conducted focus groups; analyzed focus group data; administered surveys at University of Ibadan; and revised the manuscript. RERG played a major role in data cleaning, data input, and data analysis; and drafted sections of the manuscript. NLA contributed to the data analysis and interpretation; and contributed to a section of the manuscript. SJW played a major role in the design and flow of the study instrument; performed power calculations; designed the study sampling protocol; and revised the manuscript. POS conducted focus groups; helped in the analysis of focus group data and reworking of the survey instrument; administered surveys at University of Ibadan; and revised the manuscript. MTO conducted focus groups; helped in the analysis of focus group data and reworking of the survey instrument; administered surveys at Obafemi Awolowo University; and revised the manuscript. EO conducted focus groups and helped to analyze focus group data; administered surveys at Obafemi Awolowo University; and revised the manuscript. OOA conducted focus groups; helped in the analysis of focus group data and reworking of the survey instrument; administered surveys at Covenant University; and revised the manuscript. OOO helped in the design of the study; led the logistical nightmare of getting the surveys to Nigeria and then back to the U.S.; administered surveys at Babcock University; and revised the manuscript. LS developed the Access database; performed descriptive statistics; and drafted and revised the manuscript. PSP performed data analysis; and revised the manuscript. KKO organized and conceived the entire collaboration; conceived, designed and supervised the study; and participated in the writing. All authors read and approved the final manuscript.

## Pre-publication history

The pre-publication history for this paper can be accessed here:

http://www.biomedcentral.com/1471-2458/10/610/prepub

## Supplementary Material

Additional file 1 **Women's Health and Health Behaviors Survey**. The survey instrument used in the study entitled, "Self-medication with antibiotics for the treatment of menstrual symptoms in southwest Nigeria: a cross-sectional study." Click here for file
