# Supplementary material for: Self-medication with antibiotics for the treatment of menstrual symptoms in southwest Nigeria: a cross-sectional study
Source: BMC Public Health. 2010 Oct 15;10:610. doi: 10.1186/1471-2458-10-610 (PMC2965722; doi:10.1186/1471-2458-10-610)
Supplement: Additional file 1 — Women's Health and Health Behaviors Survey. The survey instrument used in the study entitled, "Self-medication with antibiotics for the treatment of menstrual symptoms in southwest Nigeria: a cross-sectional study." [file 1471-2458-10-610-S1.PDF]

Women's Health and Health Behaviors Survey

Dear Student,

This is a survey about women's health that is being conducted by researchers at the University of Maryland, the University of Washington, the University of Ibadan, Babcok University, Covenant University and Obafemi Awolowo University.

We ask that you please accurately complete this survey. Your participation is voluntary, but it is very important because you represent many other women and the information you provide will help the improvement of health services and education for women in your area.

This survey is anonymous and all of the information you provide will remain confidential. We ask that you please *do not* write your name anywhere on the survey. Thank you for taking the time to provide this information. We greatly value your input.

If you have any questions, please contact Dr. Amy Sapkota at ars@umd.edu or Mrs. Morenike Coker at morencoker2002@yahoo.com

First we would like to ask you a few questions about your monthly menses (menstrual period).

1 During the PAST THREE MONTHS, that is SINCE DECEMBER 1, 2007, how many times have you had a menses?

_____ Times

2 During the PAST THREE MONTHS, what products have you used during your menses? (*Check all that apply*)

- ☐ Pads
- ☐ Tampons
- ☐ Cloths
- ☐ Cotton Wool
- ☐ Tissue Paper
- ☐ Other (*Please Describe*)

____

Many women experience pain or discomfort before, during, or after menses. The next few questions are about your experiences with some of these symptoms.

3 Have you EVER experienced any PAIN or DISCOMFORT associated with your menses?

- ☐ Yes
- ☐ No

4 Have you EVER seen a DOCTOR or a NURSE for discomfort or pain associated with your menses?

- ☐ Yes
- ☐ No

5 During the LAST THREE MONTHS, that is SINCE DECEMBER 1, 2007, have you experienced any PAIN or DISCOMFORT associated with your menses?

- ☐ Yes
- ☐ No

6 ***Below is a list of SYMPTOMS that women may experience before, during, or after menses.***

During the PAST THREE MONTHS, that is SINCE DECEMBER 1st, 2007, how FREQUENTLY have you experienced the following symptoms before, during, or after menses?

	Never	Rarely	Sometimes	Often	Always
Cramps	☐	☐	☐	☐	☐
Weight gain, water retention, bloating	☐	☐	☐	☐	☐
Heavy flow, heavy bleeding	☐	☐	☐	☐	☐
Headaches	☐	☐	☐	☐	☐
Pimples/Acne	☐	☐	☐	☐	☐
Aggression, moodiness	☐	☐	☐	☐	☐
Tender/painful breasts	☐	☐	☐	☐	☐
Backache, joint or muscle pain	☐	☐	☐	☐	☐
Other (*Please Describe*) ____________	☐	☐	☐	☐	☐

7 **During the PAST THREE MONTHS, HOW SEVERE was the pain or discomfort you experienced due to the following symptoms:**

	None	Mild	Moderate	Severe	Extreme
Cramps	☐	☐	☐	☐	☐
Weight gain, water retention, bloating	☐	☐	☐	☐	☐
Heavy flow, heavy bleeding	☐	☐	☐	☐	☐
Headaches	☐	☐	☐	☐	☐
Pimples/Acne	☐	☐	☐	☐	☐
Aggression, moodiness	☐	☐	☐	☐	☐
Tender/painful breasts	☐	☐	☐	☐	☐
Backache, joint or muscle pain	☐	☐	☐	☐	☐
Other *(Please Describe)* ____________	☐	☐	☐	☐	☐

Now, we will ask several questions relating to medications that women may take before, during, or after their menses.

8 **In the PAST THREE MONTHS, that is SINCE DECEMBER 1st 2007, have you taken any PAIN-RELIEVING MEDICATIONS such as Aspirin or Ibuprofen to relieve menses symptoms?**

- ☐ Yes
- ☐ No
- ☐ I don't know

9 **In the PAST THREE MONTHS, what types of pain-relieving medications have you taken to relieve any menses symptoms? Please check ALL medications that you have used, even if you only used that medication once. (*Check all that apply)***

- ☐ Aspirin
- ☐ Panadol (paracetamol)
- ☐ Panadol Extra
- ☐ Ibuprofen
- ☐ Buscopan
- ☐ Feldene (piroxicam)
- ☐ Other ____________
- ☐ I did not take any pain-relieving medications in the past 3 months

10 **Have you EVER taken ANY OTHER MEDICATIONS to treat menses symptoms?**

- ☐ Yes
- ☐ No
- ☐ I don't know

11 **Have you EVER taken ANY ANTIBIOTICS to treat menses symptoms?**

- ☐ Yes
- ☐ No
- ☐ I don't know

12 **In the PAST THREE MONTHS, that is SINCE DECEMBER 1st, 2007, have you taken any of the following SPECIFIC ANTIBIOTICS to treat menses symptoms? (Check all that apply)**

Common Trade and Drug Name(s)	Cramps	Weight gain, water retention, bloating	Heavy flow, heavy bleeding	Headaches	Pimples/Acne	Aggression, moodiness	Tender/painful breasts	Backache, joint or muscle pain	Other symptoms
**Ampicillin** "Red and Black Capsule"	☐	☐	☐	☐	☐	☐	☐	☐	☐
**Ampiclox**, (Ampicillin + Cloxacillin)	☐	☐	☐	☐	☐	☐	☐	☐	☐
**Peflotab**, Peflacine, Peloxin (Pefloxacin)	☐	☐	☐	☐	☐	☐	☐	☐	☐
**Amoxicillin**, Barbimox	☐	☐	☐	☐	☐	☐	☐	☐	☐
**Augmentin** (Amoxicillin + Clavulanate Potassium)	☐	☐	☐	☐	☐	☐	☐	☐	☐
**Ofloxacin**, Taravid, Obactin	☐	☐	☐	☐	☐	☐	☐	☐	☐
**Tetracycline**, "Red and Yellow Capsule"	☐	☐	☐	☐	☐	☐	☐	☐	☐
**Ciprotab**, Cipro-J, Ciproxin, Zefan Forte (Ciprofloxacin)	☐	☐	☐	☐	☐	☐	☐	☐	☐
**Flagyl** (Metronidazole)	☐	☐	☐	☐	☐	☐	☐	☐	☐
**Septrin** (Cotrimoxazole)	☐	☐	☐	☐	☐	☐	☐	☐	☐
**Streptomycin**	☐	☐	☐	☐	☐	☐	☐	☐	☐
**Gentamicin**, Garamycin	☐	☐	☐	☐	☐	☐	☐	☐	☐

There are many different types of medications that women may use to treat or to relieve menstrual symptoms. In the PAST THREE MONTHS are there any other medications that you have used before, during, or after menses? Please write them in below as shown in the example. If you do not know the name, please describe the medication as best you can.

	Cramps	Weight gain, water retention, bloating	Heavy flow, heavy bleeding	Headaches	Pimples/Acne	Aggression, moodiness	Tender/painful breasts	Backache, joint or muscle pain	Other symptoms
EXAMPLE: Jugyl	☐	☑	☑	☐	☐	☐	☐	☐	☐
______	☐	☐	☐	☐	☐	☐	☐	☐	☐
______	☐	☐	☐	☐	☐	☐	☐	☐	☐
______	☐	☐	☐	☐	☐	☐	☐	☐	☐

13 **During the PAST THREE MONTHS, have you taken antibiotics to treat symptoms before, during, or after menses? (*Check all that apply*)**

	Before Menses	During Menses	After Menses	I don't take Antibiotics
Cramps	☐	☐	☐	☐
Weight gain, water retention, bloating	☐	☐	☐	☐
Heavy flow, heavy bleeding	☐	☐	☐	☐
Headaches	☐	☐	☐	☐
Pimples/Acne	☐	☐	☐	☐
Aggression, moodiness	☐	☐	☐	☐
Tender/painful breasts	☐	☐	☐	☐
Backache, joint or muscle pain	☐	☐	☐	☐
Other *(Please Describe)* ______	☐	☐	☐	☐

14 **If a preferred antibiotic was not available to you, would you use another antibiotic to treat or prevent menses symptoms?**

- ☐ Yes
- ☐ No
- ☐ I do not have a preferred antibiotic.
- ☐ I do not use antibiotics.

15 **During the PAST THREE MONTHS, that is SINCE DECEMBER 1st, 2007, HOW OFTEN have antibiotics relieved the following menses symptoms:**

	Never	Rarely	Sometimes	Often	Always
Cramps	☐	☐	☐	☐	☐
Weight gain, water retention, bloating	☐	☐	☐	☐	☐
Heavy flow, heavy bleeding	☐	☐	☐	☐	☐
Headaches	☐	☐	☐	☐	☐
Pimples/Acne	☐	☐	☐	☐	☐
Aggression, moodiness	☐	☐	☐	☐	☐
Tender/painful breasts	☐	☐	☐	☐	☐
Backache, joint or muscle pain	☐	☐	☐	☐	☐
Other (*Please Describe*) ____________________	☐	☐	☐	☐	☐

16 **In the PAST THREE MONTHS, where have you obtained the antibiotics that you have used to treat menses symptoms? *(Check all that apply)***

- ☐ Local drug hawker/mobile "doctor"
- ☐ Chemist or pharmacist
- ☐ Doctor or Nurse
- ☐ Private Clinic
- ☐ Public Hospital
- ☐ Friends
- ☐ Family
- ☐ Other (*please specify*) ____________________
- ☐ I did not take antibiotics in the past 3 months.

17 **How old were you when you first started taking antibiotics to treat menses symptoms?**

_______ Years

- ☐ I don't know
- ☐ I do not take antibiotics.

18 **Who first recommended that you take antibiotics for menses symptoms?**

- ☐ Local drug hawker/mobile "doctor"
- ☐ Chemist or pharmacist
- ☐ Doctor or Nurse
- ☐ Friend
- ☐ Mother
- ☐ Sister
- ☐ Other (*please specify*) ____________________
- ☐ I don't know.
- ☐ Nobody has ever recommended that I take antibiotics.

This last section of the questionnaire asks for some additional information about you. Because you represent many women, knowing more about your background can help us to better understand your experiences. Some questions may also look a bit different because we would like for you to tell us about your experiences and your opinions in your own words.

19 **How old are you?**

- ☐ Less than 17 years old
- ☐ 17-20
- ☐ 21-24
- ☐ 25-29
- ☐ 30-34
- ☐ 35-39
- ☐ 40 or above

20 **As of this semester, what is your current classification at the University?**

- ☐ Preliminary
- ☐ 100 Level
- ☐ 200 Level
- ☐ 300 Level
- ☐ 400 Level
- ☐ 500 Level
- ☐ 600 Level
- ☐ 700 Level or above

21 **Below is a list of general areas of study. Please select your area(s) of study at the University.** ***Check all that apply. If your area of study is not in the list below, please check "Other" and write in your area of study.***

- ☐ Agriculture
- ☐ Arts
- ☐ Business Administration
- ☐ Education
- ☐ Environmental Science/Studies
- ☐ Engineering
- ☐ Law
- ☐ Medicine or Nursing
- ☐ Pharmacy
- ☐ Science (Biological, Physical, Chemical)
- ☐ Social Science
- ☐ Theology/Religious Studies
- ☐ Veterinary Medicine
- ☐ Other (*Please Specify*)

____

22 **This survey contains a lot of questions about different types of medication that women may take to treat menstrual symptoms. Several questions are about ANTIBIOTICS. What do you know about ANTIBIOTICS? Have you heard about using specific antibiotics or other medication as treatment for menstrual symptoms? From whom did you hear this? Have you participated in any other survey on menstrual symptom treatments and/or antibiotics use before?**

____
____
____
____
____
____
____
____
____
____
____
____

The last three questions on this survey ask you for information that you may find particularly sensitive. ALL information you provide on this survey is anonymous and confidential. These questions may be difficult to answer but they are also very important.

23 **Are you currently sexually active?**

- ☐ Yes
- ☐ No

24 **What is your marital status?**

- ☐ Single, never married
- ☐ Engaged
- ☐ Married
- ☐ Separated
- ☐ Divorced
- ☐ Widowed

25 **Do you have any children?**

- ☐ Yes
- ☐ No

(IF YES TO 24) **How many children do you have?**

_____ Children

THANK YOU very much for taking the time to complete this survey. Your input is very important and will help us to improve women's health education and services.
